# The Alzheimer’s β-secretase BACE1 localizes to normal presynaptic terminals and to dystrophic presynaptic terminals surrounding amyloid plaques

**DOI:** 10.1007/s00401-013-1152-3

**Published:** 2013-07-03

**Authors:** Patty C. Kandalepas, Katherine R. Sadleir, William A. Eimer, Jie Zhao, Daniel A. Nicholson, Robert Vassar

**Affiliations:** 1 Department of Cell and Molecular Biology, The Feinberg School of Medicine, Northwestern University, 300 East Superior Street, Tarry 8-713, Chicago, IL 60611-3006 USA; 2 Department of Neurological Sciences, Rush University Medical Center, 1750 West Harrison Street, Jelke Building, Suite 1474, Chicago, IL 60612 USA; 3 Department of Neuroscience, GlaxoSmithKline, Inc., 277 Niudun Road, Pudong Shanghai, China

**Keywords:** Alzheimer’s disease, BACE1, β-Secretase, Aβ, Amyloid plaque, Dystrophic neurite, Lysosome, Autophagy

## Abstract

β-Site amyloid precursor protein (APP) cleaving enzyme-1 (BACE1) is the β-secretase that initiates Aβ production in Alzheimer’s disease (AD). BACE1 levels are increased in AD, which could contribute to pathogenesis, yet the mechanism of BACE1 elevation is unclear. Furthermore, the normal function of BACE1 is poorly understood. We localized BACE1 in the brain at both the light and electron microscopic levels to gain insight into normal and pathophysiologic roles of BACE1 in health and AD, respectively. Our findings provide the first ultrastructural evidence that BACE1 localizes to vesicles (likely endosomes) in normal hippocampal mossy fiber terminals of both non-transgenic and APP transgenic (5XFAD) mouse brains. In some instances, BACE1-positive vesicles were located near active zones, implying a function for BACE1 at the synapse. In addition, BACE1 accumulated in swollen dystrophic autophagosome-poor presynaptic terminals surrounding amyloid plaques in 5XFAD cortex and hippocampus. Importantly, accumulations of BACE1 and APP co-localized in presynaptic dystrophies, implying increased BACE1 processing of APP in peri-plaque regions. In primary cortical neuron cultures, treatment with the lysosomal protease inhibitor leupeptin caused BACE1 levels to increase; however, exposure of neurons to the autophagy inducer trehalose did not reduce BACE1 levels. This suggests that BACE1 is degraded by lysosomes but not by autophagy. Our results imply that BACE1 elevation in AD could be linked to decreased lysosomal degradation of BACE1 within dystrophic presynaptic terminals. Elevated BACE1 and APP levels in plaque-associated presynaptic dystrophies could increase local peri-plaque Aβ generation and accelerate amyloid plaque growth in AD.

**Electronic supplementary material:**

The online version of this article (doi:10.1007/s00401-013-1152-3) contains supplementary material, which is available to authorized users.

## Introduction

Alzheimer’s disease (AD) is characterized by the presence of neurofibrillary tangles and cerebral amyloid plaques composed of the 40–42 amino acid β-amyloid peptide (Aβ; reviewed in Sisodia and Hyslop [98]). Much evidence implicates Aβ in the pathogenesis of AD (reviewed in [102]). Aβ is generated from the sequential proteolysis of amyloid precursor protein (APP) by the enzymes β-secretase and γ-secretase (reviewed in [21, 122]). β-Secretase initiates the cleavage of APP and has been identified as the transmembrane aspartic protease beta-site APP cleaving enzyme 1 (BACE1) [40, 64, 96, 106, 118]. As the initiator enzyme of the amyloidogenic pathway, BACE1 is a prime therapeutic target for reducing cerebral Aβ levels, and several BACE1 inhibitor drug candidates are currently in clinical trials for AD.

Elucidating the physiological functions of BACE1 is essential for predicting potential mechanism-based toxicities associated with BACE1 inhibition as a therapeutic approach for AD. BACE1-null mice display complex neurological phenotypes, including growth retardation [24], memory deficits [57, 77, 78], hypomyelination [37, 113], seizures [33, 39, 50], axon guidance defects [13, 32, 85], and schizophrenia-like behaviors [91]. These BACE1^−/−^ phenotypes likely reflect the functions of a diverse array of BACE1 substrates that include Golgi-localized membrane-bound α2,6-sialyltransferase [49], P-selectin glycoprotein ligand-1 [63], APP and the APP homolog proteins APLP1 and APLP2 [25, 61, 79], low density lipoprotein receptor-related protein [108], the voltage-gated sodium channel β2 subunit (Na_v_β_2_) [46, 47, 114], neuregulin-1 (NRG1) [37, 113], neuregulin-3 (NRG3) [36], and Close Homolog of L1 (CHL1) [32, 55, 123], among others [55, 123]. Additional as yet unknown BACE1 substrates are likely to exist and their identification will provide further insight into the biological functions of BACE1.

Understanding the subcellular localization of BACE1 may provide important clues as to the identities of key BACE1 substrates and the physiological functions of substrate processing by BACE1, especially in neurons of the brain where therapeutic inhibition of BACE1 will be targeted. Previously, using immunohistochemistry with a mono-specific anti-BACE1 antibody, we reported that BACE1 accumulates in swollen presynaptic neuronal structures that surround amyloid plaques in AD and APP transgenic brains [121]. In that and another study, we also observed that BACE1 levels in the normal mouse brain were highest in axon terminal fields, especially within presynaptic terminals of the hippocampal mossy fiber pathway in CA3 [32, 121]. These results suggest that BACE1 has normal and aberrant presynaptic roles in non-demented and Alzheimer disease brains, respectively.

To further characterize the localization of BACE1 at both the light and electron microscopic levels and thereby infer potential normal and abnormal activities of BACE1 in the brain, we performed immunofluorescence confocal microscopy and immunogold electron microscopy (EM) of mossy fiber terminal regions in the hippocampal CA3 subregion in non-transgenic and APP transgenic (5XFAD) mouse brains. The high level of BACE1 in the mossy fiber rendered it an excellent model to investigate the subcellular localization of BACE1 in vivo. Our findings provide the first unequivocal demonstration that BACE1 localizes to vesicles in large presynaptic mossy fiber terminals within CA3 in both normal and AD model mouse brains. In some cases, BACE1-immunopositive vesicles were located near active zones, implying an important but as yet undetermined function of BACE1 at the synapse. Moreover, BACE1 was also enriched within a distinct subtype of dystrophic presynaptic neurite that surrounds the amyloid plaque in the APP transgenic brain. These BACE1-positive presynaptic dystrophies tended to contain fewer large electron-dense multilamellar autophagosomes. This was true for neuritic dystrophies observed within the hippocampus as well as in the cerebral cortex. Importantly, we observed that accumulations of BACE1 and APP co-localized in presynaptic dystrophies, implying increased BACE1 processing of APP in these abnormal peri-plaque regions. In addition, BACE1 partially co-localized with transferrin receptor, suggesting that BACE1 accumulates in endosomes of dystrophic terminals. In primary cortical neuron cultures, treatment with the lysosomal protease inhibitor leupeptin resulted in increased BACE1 levels; however, exposure of neurons to the autophagy inducer trehalose did not reduce BACE1 levels, suggesting that BACE1 is degraded in the lysosomal, but not the autophagic, pathway. Though the precise mechanism of BACE1 accumulation within aberrant presynaptic dystrophies in the APP transgenic brain is currently unknown, our data as well as other published reports imply a link to decreased lysosomal degradation of BACE1 within dystrophic axon terminals. An increase in BACE1 level in plaque-associated dystrophic presynaptic terminals, in conjunction with APP accumulation in these neuritic dystrophies [19, 20], may elevate local peri-plaque Aβ generation and exacerbate the progression of amyloid pathology in AD.

## Materials and methods

### Animals

5XFAD mice have been described previously [75] and were maintained on a B6/SJL F1 hybrid background. BACE1^−/−^ mice on a C57/B6 background were obtained from Jackson Laboratories (strain #006554). Non-transgenic age-matched B6/SJL or C57/B6 mice were used as controls. Procedures were performed with Northwestern University IACUC approval.

### Tissue collection for immunoblots and confocal microscopy

Mice were perfused with cold phosphate buffered saline (PBS) containing protease and phosphatase inhibitors [20 μg/ml phenylmethylsulfonyl fluoride (PMSF, Sigma), 500 ng/ml leupeptin (MP Biomedicals), 20 μM sodium orthovanadate (MP Biomedicals), 10 μM dithiothreitol (DTT, Sigma)]. A hemibrain from each mouse was dissected into hippocampus and cortex and snap-frozen separately for biochemical analyses; the other hemibrain was drop-fixed in 4 % paraformaldehyde overnight at 4 °C and cryopreserved in 30 % (w/v) sucrose/PBS at 4 °C for histology. Fixed cryopreserved human Braak stage V–VI brain tissues (entorhinal cortex and superior temporal gyrus) from three AD cases were obtained from the Cognitive Neurology and AD Center at Northwestern University.

### Immunoblot analysis

5XFAD and non-transgenic (5 each) hippocampi were individually homogenized in 1 % Triton X-100/PBS with 1× protease inhibitor (Calbiochem) and 1× Halt Phosphatase Inhibitor cocktail (Thermo Scientific). Protein concentration was quantified by BCA (Thermo Scientific). Hippocampal homogenates (20 μg) and primary neuron lysates (10 μg) were separated by 12 % Tris–Glycine or 4–12 % Bis–Tris SDS-PAGE and transferred onto 0.45 μm PVDF membranes (Millipore) that were subsequently Ponceau stained, scanned, and probed with the following antibodies recognizing BACE1 (1:1000; BACE–Cat1 [121]), LC3B (1:1,000 or 1:4,000; Cell Signaling #3868), and β-III-tubulin (TuJ1) (1:10,000; gift of Dr. Lester Binder). Membranes were then washed with TBST and incubated with the appropriate HRP-conjugated secondary antibodies (1:10,000; Vector Laboratories), washed again and visualized using Luminata Crescendo (Millipore). Signals were quantified using a Kodak Image Station 4000R. Signal intensities were normalized to tubulin or ponceau staining as indicated.

### Mouse immunofluorescence confocal microscopy

Free-floating hemibrain coronal sections (30 μm) from 5XFAD and non-transgenic mice (2–3 each) were cut on a freezing microtome, washed in TBS, and blocked in 5 % goat or donkey serum. Sections were incubated at 4 °C overnight on a shaker with the following primary antibodies: mouse monoclonal anti-BACE1 (1:250; BACE–Cat1 [121]) or rabbit monoclonal anti-BACE1 (1:250; Epitomics #EPR3956), goat polyclonal anti-synaptophysin (1:250 mouse brain, 1:50 human brain; R&D Systems #AF5555), goat polyclonal anti-APP (1:500; Karen, gift of Dr. Virginia Lee), rat monoclonal anti-transferrin receptor (1:500; Abcam #ab60344), rat monoclonal anti-LAMP1 (1:500; Abcam #ab25245), mouse monoclonal anti-β-III-tubulin (TuJ1) (1:100; gift of Dr. Lester Binder), chicken polyclonal anti-microtubule-associated protein 2 (MAP2) (1:250; Abcam #ab5392), mouse monoclonal anti-neurofilament NFT160 (1:250; Sigma #N5264), rabbit monoclonal anti-LC3B (1:500; Cell Signaling #3868). Sections treated with Epitomics anti-BACE1 primary antibody were incubated for 2 h at 37 °C on a shaker. Following primary antibodies, sections were washed in TBS and incubated with Alexa Fluor secondary antibodies at 1:1,000 (donkey anti-mouse or rabbit-488 or 594; goat anti-mouse or rabbit-488 or 594) and DAPI (Invitrogen), washed, mounted on charged slides and cover-slipped using ProLong Gold (Invitrogen). Images were captured on Nikon (Tokyo, Japan) A1R or Zeiss LSM 510 laser scanning confocal microscopes.

### Human immunofluorescence confocal microscopy

Human AD sections were processed using the same method as mouse, with the addition of antigen retrieval and autofluorescence reduction steps. Free-floating sections (40 μm) from 3 Braak stage V–VI AD brains were washed in TBS and incubated for 1 h in 16 mM glycine on a shaker. After TBS washes, sections underwent antigen retrieval using 0.1 M sodium citrate, pH 9.0, for 1.5 h at 80–90 °C, TBS washed, and incubated in KMnO_4_ until brown. Sections were washed with DI water, treated with 0.5 % oxalic acid and 0.5 % K_2_S_2_O_5_ to remove brown color, washed again, and incubated on a shaker for 30 min in 0.25 % NaBH_4_. Following a 0.25 % Triton/TBS wash, sections were blocked and incubated with primary antibodies as in the mouse immunofluorescence procedure.

### Pre-embedding ultrasmall silver-intensified immunogold electron microscopy

Two to three adult (4–14 months) 5XFAD, non-transgenic (C57Bl6/SJL), and BACE1^−/−^ mice were anesthetized with isoflurane (Isothesia, Butler), transcardially perfused with 0.9 % saline followed by 50 ml ice-cold acidic fixative (2 % paraformaldehyde, 1 % glutaraldehyde in 0.1 M sodium acetate, pH 6.0), then slowly perfused with ice-cold basic fixative (2 % paraformaldehyde, 1 % glutaraldehyde in 0.1 M sodium borate buffer, pH 9.0) for 1 h. Brains were removed, placed in ice-cold basic fixative on a shaker at 4 °C overnight. The following day brains were bisected, washed 3 × 20 min in TBS, and cut into 70 μm coronal sections on a vibratome. Sections were washed in TBS 5 × 5 min, treated with 1 % NaBH_4_ in TBS for 30 min, washed in TBS 5 × 1 min and incubated in blocking solution (10 % NGS in TBS) for 30 min followed by incubation in primary antibody overnight at 4 °C. Primary antibody [rabbit monoclonal anti-BACE1 (1:500; Cell Signaling #5606 or Epitomics #EPR3956); mouse monoclonal anti-β-III-tubulin (TuJ1) (1:100; gift from Dr. Lester Binder); rabbit polyclonal anti-synaptophysin (1:500; Millipore #AB9272)] was diluted in 2 % NGS + 0.1 or 0.5 % Triton X-100 in TBS. Sections were washed 1 × 5 min in incubation buffer and 10 × 5 min in TBS, then incubated in secondary blocking buffer [2 % NGS + 1 % BSA + 0.3 % cold water fish skin gelatin (CWFSG) in TBS] for 1 h followed by incubation in Ultra Small Immunogold (Aurion) anti-rabbit secondary antibody at 1:100 in 2 % NGS + 1 % BSA-C + 0.3 % CWFSG at 4 °C for ~40 h. Sections were washed in incubation buffer 1 × 5 min, TBS 6 × 10 min, PBS 2 × 5 min then fixed in 2 % glutaraldehyde in PBS for 1 h, followed by washes 2 × 5 min in PBS, 4 × 10 min in TBS, and 3 × 10 min in enhancement conditioning solution (ECS). Sections were then incubated in R-Gent SE-EM Plus enhancement mixture for 90 min and washed in ECS 4 × 10 min, TBS 2 × 10 min, and PBS 2 × 10 min, osmicated with 0.4 % OsO_4_ in PBS for 15 min and rinsed in PBS 3 × 10 min and dH_2_O 2 × 5 min. Sections were stained in 1 % aqueous uranyl acetate for 10 min, rinsed 3 × 10 min in dH_2_O, dehydrated in graded ethanol and propylene oxide, infiltrated with 1:1 araldite:propylene oxide overnight at room temperature, followed by flat embedding between aclar sheets and curing for 48 h at 60 °C. Regions of interest were subdissected and re-embedded in Araldite and cured overnight at 60 °C. 500-nm-thick histological sections were cut by ultramicrotome and stained with toluidine blue to confirm the presence of the stratum lucidum. Serial ultrathin sections (63 nm) were cut with a diamond knife, placed onto formvar-coated slotted grids, stained with uranyl acetate–lead citrate (for 15 and 10 min, respectively), washed in ultrapure dH_2_O, and allowed to dry at room temperature. Images were taken with a JEOL 1200EX electron microscope (JEOL Ltd., IL, USA) at a magnification of 7500–20,000×. Electron micrographs from 8 to 30 serial sections containing mossy fiber terminals or dystrophic neurites were obtained between 1 and 8 microns from the tissue surface (i.e., the surface of the 70-μm section that was immunogold-labeled) [111]. EM reagents were from Electron Microscopy Sciences (Hatfield, PA) unless otherwise noted.

### Conventional electron microscopy

One adult 5XFAD, C57/Bl6, and BACE1^−/−^ mouse was anesthetized with isofluorane (Isothesia, Butler), perfused transcardially with 0.12 M PBS (pH 7.4) for 1 min, then a dilute aldehyde mixture (1 % paraformaldehyde, 1.25 % glutaraldehyde, 0.02 mM CaCl_2_ in 0.1 M sodium cacodylate buffer) for 30 min, and a concentrated aldehyde mixture (2 % paraformaldehyde, 2.5 % glutaraldehyde, 0.04 mM CaCl_2_ in 0.1 M sodium cacodylate buffer) for 10 min. Brains were removed and placed in ice-cold concentrated fixative on a shaker at 4 °C overnight. The following day brains were bisected, rinsed 3 × 20 min in 0.12 M TBS and cut into 70 μm coronal sections as above. Sections were washed in 0.12 M phosphate buffer (PB) 3 × 10 min at 4 °C, treated with 2 % OsO_4_ in 0.12 M PB for 1 h at 4 °C, and washed 3 × 10 min in 0.12 M PB. The tissue was then dehydrated in graded ethanols and propylene oxide, infiltrated with 1:1 araldite:propylene oxide, flat embedded between aclar sheets and cured for 48 h at 60 °C. Regions of interest were subdissected and re-embedded as above. Serial ultrathin sections (65 nm) were cut, placed onto formvar-coated slotted grids, then stained with uranyl acetate–lead citrate (15 and 10 min, respectively) and rinsed in ultrapure dH_2_O. Images (7500–20,000×) were taken with a JEOL 1200EX electron microscope (JEOL Ltd., IL, USA) from 10–30 serial sections.

### Primary neuron culture

Cortical neurons were isolated from day 15.5–16.5 C57/B6 mouse embryos via dissociation at 37 °C in 0.25 % trypsin. Neurons were plated at a density of 750,000 cells per well in poly-l-lysine coated 12-well plates containing neurobasal media supplemented with 2 % B-27, 500 μM glutamine, 10 % horse serum and 2.5 μM glutamate. After 2–3 h, the media was replaced with neurobasal media with 2 % B-27, 500 μM glutamine, and 2.5 μM glutamate. After 3 DIV, media was replaced with neurobasal media with 2 % B-27 and 500 μM glutamine. All cell culture reagents were from Invitrogen. After 6 DIV, neurons were exposed to 20 μM leupeptin (MP Biomedicals), 150 mM trehalose (Sigma), or both for 24 or 48 h. In a separate experiment, neurons were treated with 100 nM bafilomycin (Sigma) for 4 h. Neurons were lysed in RIPA buffer (150 mM NaCl, 1 % IGEPAL CA-630, 0.5 % sodium deoxycholate, 0.1 % SDS, 50 mM Tris pH 8, 1 mM PMSF) with 1× protease inhibitors (Calbiochem) and 1× Halt Phosphatase Inhibitor Cocktail (Thermo Scientific). Lysates were centrifuged at 10,000 rpm, 4 °C, 10 min and the supernatant protein was quantified by BCA (Thermo Scientific).

### Statistical analyses

Densitometric analyses of immunoblots were performed using Kodak 1D 3.6 image analysis software. Statistical differences for immunoblot experiments were determined using two-tailed students *t* test or ANOVA (GraphPad Software, Inc., San Diego, CA). Graphed data are presented as the mean ± SEM, and *p* < 0.05 was considered significant.

## Results

### BACE1 is localized in presynaptic terminals of normal brain at the ultrastructural level

**Fig. 1:**
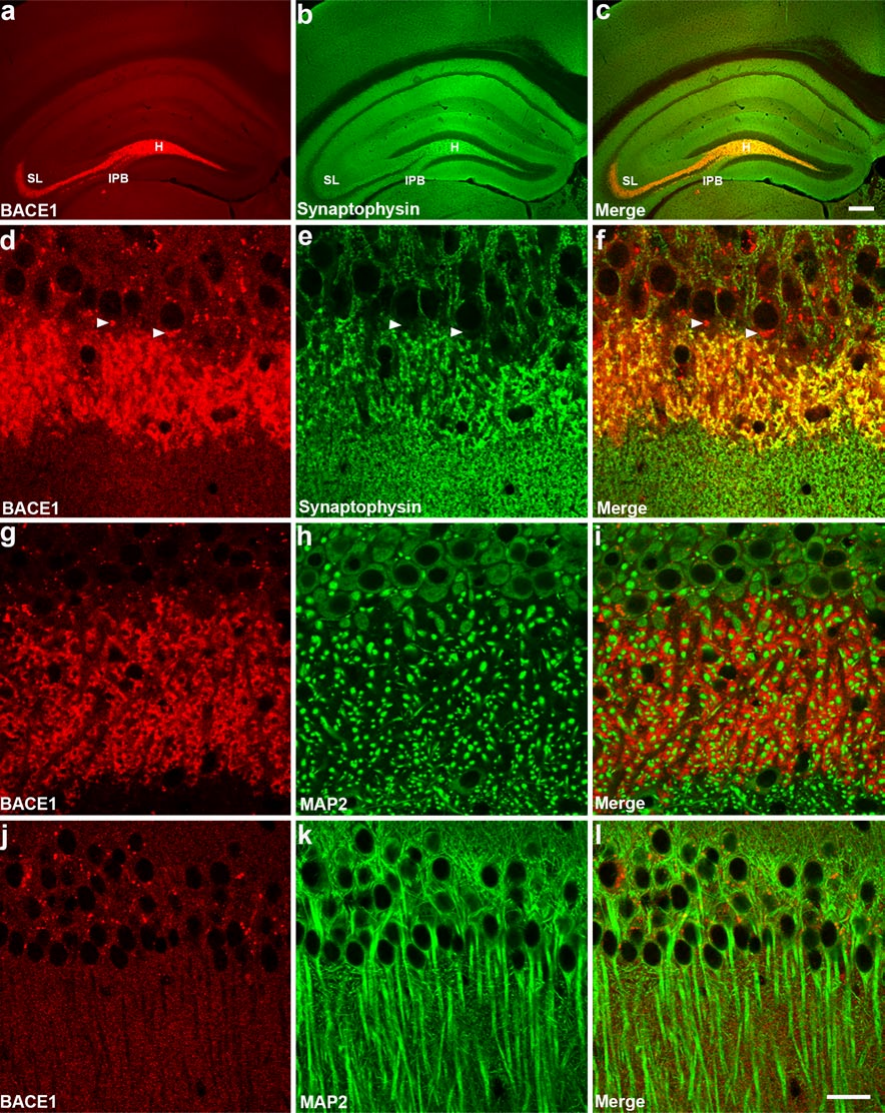
BACE1 is localized within presynaptic terminals at the light microscopic level. Representative images of coronal brain sections from 2- to 3-month-old wild-type mice co-stained with BACE1 (*red*) and synaptophysin or MAP2 (*green*) antibodies and imaged by laser scanning confocal microscopy. **a**–**c** At low magnification, BACE1 immunoreactivity is observed in the hilar region of the dentate gyrus (H), and in the infrapyramidal bundle (IPB) and stratum lucidum (SL) of the hippocampal mossy fiber pathway, where extensive co-labeling with synaptophysin also occurred, denoting presynaptic localization of BACE1 within these brain regions. **d**–**f** Higher magnification of BACE1 and synaptophysin immunoreactivity within the stratum lucidum of the wild-type mouse shown in **a**–**c**. BACE1 and synaptophysin signals significantly co-localize within mossy fiber terminals in **f**. Note the punctate BACE1 signals within neuronal soma (examples indicated by *white arrowheads*), which do not overlap with synaptophysin immunoreactivity and likely represent TGN and endosomes. **g**–**l** BACE1 immunoreactivity does not overlap with that of the somatodendritic marker MAP2 (*green*) within the stratum lucidum in CA3 (**g**–**i**) or the stratum radiatum in CA1 (**j**–**l**). *Scale bar* **a**–**c**, 200 μm; **d**–**l**, 25 μm

Previously, we generated a mono-specific anti-BACE1 antibody (BACE–Cat1) that does not cross-react with any other protein in the brain [121]. Initial BACE1 immunohistochemistry results using BACE–Cat1 suggested that the highest levels of BACE1 in the brain were located in stratum lucidum of the CA3 hippocampal subregion [121]. Earlier reports indicated that BACE1 is predominantly expressed in neurons [40, 96, 106], although these studies did not determine the subcellular localization of BACE1 in neurons of the normal brain. To investigate BACE1 localization, we co-labeled coronal brain sections from wild-type mice with BACE–Cat1 and antibodies against the presynaptic terminal marker synaptophysin or the somatodendritic marker microtubule-associated protein 2 (MAP2) and performed immunofluorescence confocal microscopy (Fig. 1). We observed that the vast majority of BACE1 immunostaining co-localized with synaptophysin labeling (Fig. 1c, f) and displayed a punctate pattern that represents the large mossy fiber terminals (MFTs/giant boutons) of dentate gyrus granule cell axons within the stratum lucidum region of CA3 (Fig. 1d). In addition, faint punctate immunolabeling of BACE1 co-localized with that of synaptophysin in the hippocampus (Fig. 1d–f) and throughout the rest of the brain (not shown), indicating that BACE1 localizes generally to presynaptic terminals in the CNS. A small proportion of BACE1 puncta was also seen in neuronal soma in the pyramidal layers of CA1 and CA3, which likely represents BACE1 localization within the trans-golgi network (TGN) or endosomal compartments of cell bodies (Fig. 1d, white arrowheads). BACE1 puncta within cell bodies did not co-localize with synaptophysin (Fig. 1d–f). In contrast to BACE1 co-localization with synaptophysin, we did not detect co-localization of BACE1 and MAP2 in either CA3 (Fig. 1g–i) or CA1 (Fig. 1j–l). Taken together, our data and other published reports [57, 93] indicate that a large proportion of endogenous BACE1 in the brain is localized within presynaptic neuronal terminals, but little if any BACE1 is present in postsynaptic areas other than the soma.

**Fig. 2:**
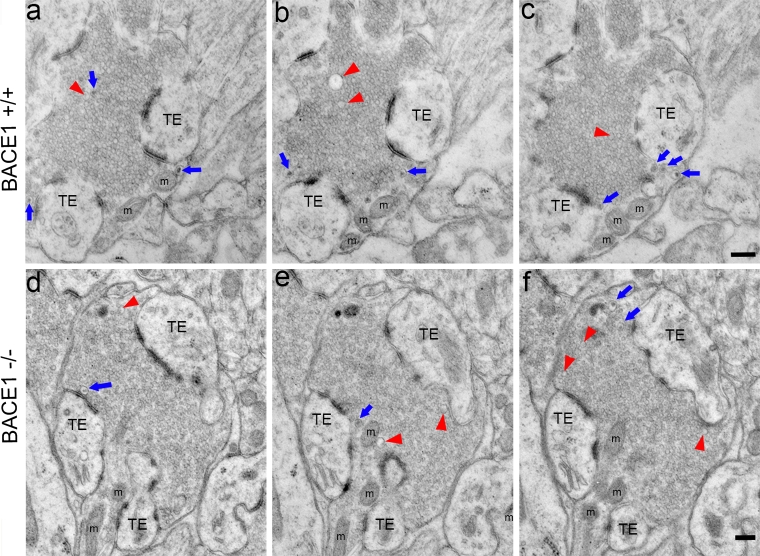
BACE1^−/−^ mossy fiber terminals have normal ultrastructure. Serial ultrathin sections of the stratum lucidum from 5-month-old BACE1^+/+^ (**a**–**c**) and 11-month-old BACE1^−/−^ (**d**–**f**) mice were imaged by electron microscopy. Shown are representative single presynaptic mossy fiber terminals (MFTs) contacting postsynaptic thorny excrescences (TE) with clearly recognizable synapses (bands of electron-dense material). Note the compact pool of synaptic vesicles distributed throughout the terminals. A high abundance of small, clear presynaptic vesicles can be resolved, as well as a lower abundance of large clear vesicles (*red arrowheads*) and dense-core vesicles (*blue arrows*). Mitochondria (m) of various shapes and sizes are located near the plasma membranes of the MFTs. No morphological differences in MFTs are evident between BACE1^+/+^ and BACE1^−/−^ mice. *Scale bars* 200 nm

Although our BACE1 immunofluorescence confocal microscopy results indicated that BACE1 is predominantly presynaptic, light microscopy lacks the resolution to determine the precise subcellular localization of BACE1 in the axon terminal. Therefore, we performed serial section pre-embedding silver-intensified ultrasmall BACE1 immunogold electron microscopy (immuno-EM), as well as conventional EM. We focused specifically on hippocampal MFTs within stratum lucidum, which serve as excellent models of presynaptic terminals for our BACE1 studies: they are large, have easily identifiable morphology [2, 15, 26, 88, 112], and exhibit the highest concentration of BACE1 in the brain [121]. The ultrastructural features of MFTs allow for unequivocal identification by EM [2, 15, 26, 88, 112]. By means of conventional EM, CA3 presynaptic terminals from wild-type (BACE1^+/+^, Fig. 2a–c) and BACE1^−/−^ (Fig. 2d–f) brains were morphologically indistinguishable. BACE1^+/+^ and BACE1^−/−^ MFTs contained large vesicle pools densely packed with small, clear synaptic vesicles, some large clear vesicles, and a few intermingled dense-core vesicles. Mitochondria were situated along the periphery of the terminals, and thorny excrescences of CA3 pyramidal cell dendrites invaginated the respective terminals with distinct postsynaptic densities. Thus, genetic ablation of BACE1 did not have an obvious effect on vesicle organization or gross morphology of axon terminals within the CA3 subregion of the murine hippocampus. This data, coupled with the absence of BACE1 immunoreactivity (Suppl. Fig. 1b, d), rendered the BACE1^−/−^ mouse a good negative control for our immuno-EM studies.

Our initial attempts at defining BACE1 localization by immuno-EM proved futile, as our BACE–Cat1 antibody was not amenable to the fixation protocol used to preserve tissue for electron microscopic analysis. However, with the recent development of superior commercial anti-BACE1 antibodies, we were able to identify—with ultrastructural resolution—the precise subcellular location of BACE1 within the murine brain. To accomplish this, we incubated coronal sections of mouse hippocampus with a rabbit monoclonal anti-BACE1 antibody and goat anti-rabbit IgG conjugated to ultrasmall gold particles followed by silver enhancement, as previously described [60, 111]. As expected [57, 121], immunogold particles for BACE1 were concentrated within the hilar region of the dentate gyrus, the infrapyramidal bundle, and stratum lucidum of CA3, an exact match to the previously observed BACE1 immunofluorescence labeling pattern (Suppl. Fig. 1a, c). As a negative control to demonstrate antibody specificity, hippocampal sections from BACE1^−/−^ mice treated with the anti-BACE1 antibody lacked BACE1 immunoreactivity using either the immunofluorescence or immunogold staining protocol (Suppl. Fig. 1b, d).

**Fig. 3:**
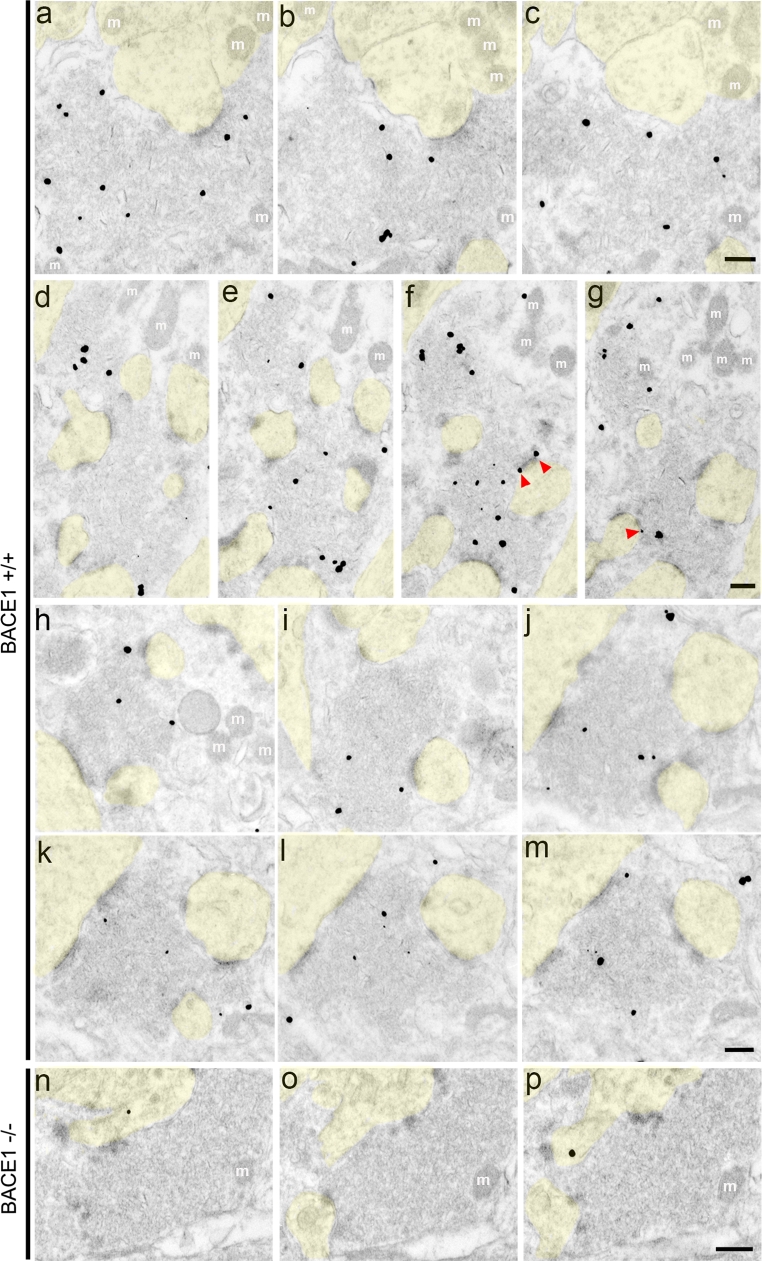
BACE1 is localized within mossy fiber terminals at the electron microscopic level. Coronal brain sections from 4- to 14-month-old BACE1^+/+^ (**a**–**m**) and BACE1^−/−^ (**n**–**p**) mice were processed for BACE1 pre-embedding silver-intensified ultrasmall immunogold and then serial ultrathin sections of the stratum lucidum were imaged by electron microscopy. **a**–**p** Representative serial images of single mossy fiber terminals. BACE1 immunoreactivity is clearly enriched within presynaptic terminals, although there is heterogeneity in the abundance of gold particles within a given terminal as well as distance from synapses (bands of electron-dense material). In some cases, BACE1 immunogold particles are located in close proximity to an active zone (**f**, **g** *red arrowheads*). Postsynaptic regions (dendrites and thorny excrescences, shaded *yellow*) contain little to no BACE1 immunoreactivity. **n**–**p** BACE1 immunogold particles are absent from presynaptic terminals in BACE1^−/−^ mice, although rare background particles (in this case, postsynaptic) are present. *Scale bars* 200 nm

When we used electron microscopy to localize BACE1 immunogold particles within the stratum lucidum of BACE1^+/+^ mice, we observed robust BACE1 immunoreactivity within the large presynaptic terminals of the mossy fibers that synapse onto the thorny excrescences and proximal dendrites of CA3 pyramidal neurons (Fig. 3a–m). We detected heterogeneity amongst the level and spatial distribution of BACE1 immunogold particle labeling throughout different mossy fiber terminal fields. Importantly, the vast majority of BACE1 immunogold was localized over areas that exhibited the densely packed synaptic vesicles that we observed in MFTs by conventional EM (Fig. 2), although there were no obvious morphological characteristics distinguishing immunopositive from immunonegative vesicles. Occasionally, gold particles were projected onto vesicles very near the active zone of a synapse (Fig. 3f, g, red arrowheads). Though the vast majority of immunogold labeling was presynaptic, a very small number of postsynaptic gold particles were noted that we suspected might have represented background. This suspicion was supported by the presence of occasional gold particles observed in postsynaptic elements within BACE1^−/−^ brains, which otherwise lacked BACE1 immunogold labeling within presynaptic compartments (Fig. 3n–p). Taken together, our BACE1 immunofluorescence and immuno-EM results unequivocally demonstrate that endogenous BACE1 is predominantly localized to the presynaptic terminal in the normal murine hippocampus. In addition, our data suggest that BACE1 is present in vesicles, some in close proximity to synaptic active zones, although the exact identity of BACE1-containing vesicles has yet to be determined.

### BACE1 is localized within endocytic vesicles of dystrophic presynaptic terminals in the APP transgenic brain at the ultrastructural level

**Fig. 4:**
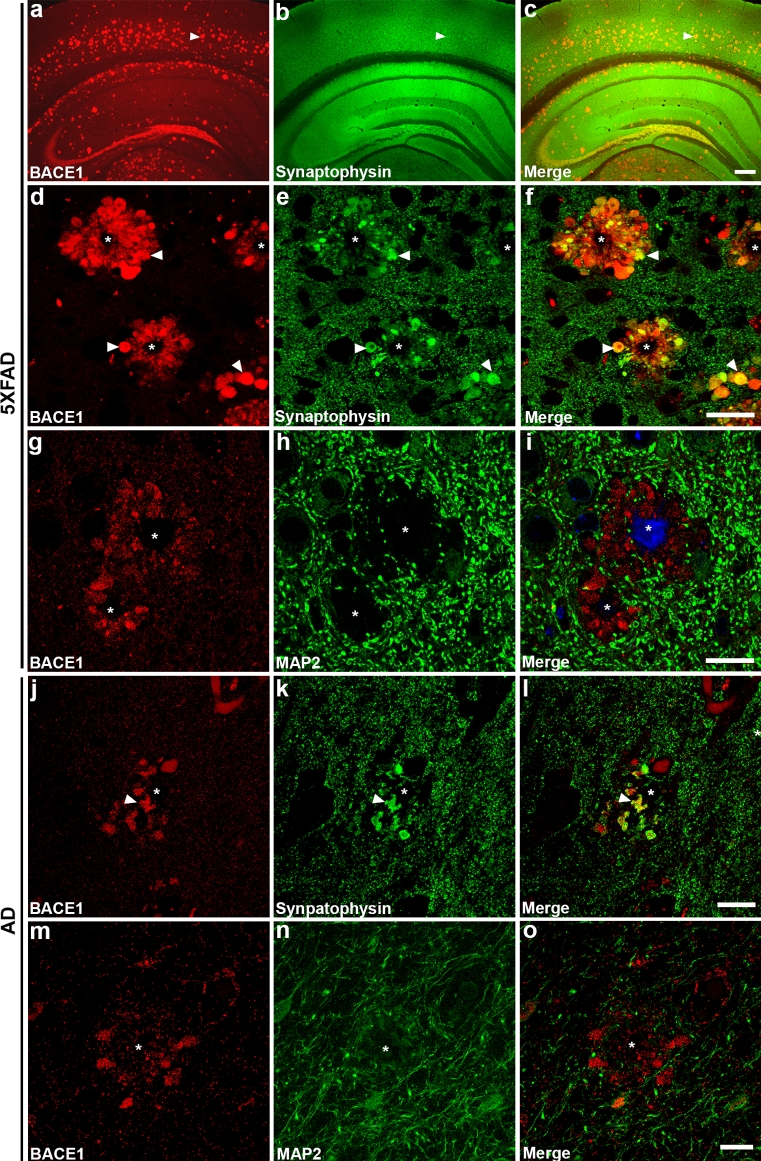
BACE1 accumulates presynaptically around amyloid plaques in 5XFAD transgenic mouse and human AD brains. Representative images of sections from 5XFAD transgenic mouse (~6 months old) or human AD brains co-stained with BACE1 (*red*) and synaptophysin or MAP2 (*green*) antibodies and imaged by laser scanning confocal microscopy. **a**–**c** At low magnification, in addition to normal BACE1 localization in hippocampal mossy fibers, BACE1 immunoreactivity (*red*) displays a plaque-like staining pattern that overlaps with synaptophysin (*green*) immunoreactivity in the 5XFAD brain (*white arrowheads* indicate representative plaque). **d**–**f** Higher magnification images showing extensive co-localization of BACE1 and synaptophysin around amyloid plaques (*white asterisks*) in 5XFAD brain, demonstrating accumulation of BACE1 in abnormal presynaptic structures, likely dystrophic terminals (*white arrowheads* indicate representative dystrophies with BACE1-synaptophysin co-localization). **g**–**i** No overlap between BACE1 and the somatodendritic marker, MAP2 (*green*), is apparent in dystrophic structures surrounding plaque cores stained with thioflavin S (*blue*). Superior temporal gyrus or entorhinal cortex brain sections of Braak stage V–VI human AD brains were co-stained with antibodies against BACE1 (*red*) and synaptophysin (**j**–**l**) or MAP2 (**m**–**o**) (*green*). BACE1 immunoreactivity overlaps with that of synaptophysin (**j**–**l**) but not MAP2 (**m**–**o**) within some of the abnormal structures near plaques in human AD (e.g., *white arrowheads*). Although immunostaining was somewhat weaker in AD compared to 5XFAD brain sections, both exhibited qualitatively similar patterns of presynaptic BACE1 accumulation around plaques. *Scale bars* **a**–**c**, 200 μm; **d**–**f**, **j**–**o**, 20 μm; **g**–**i**, 25 μm

**Fig. 5:**
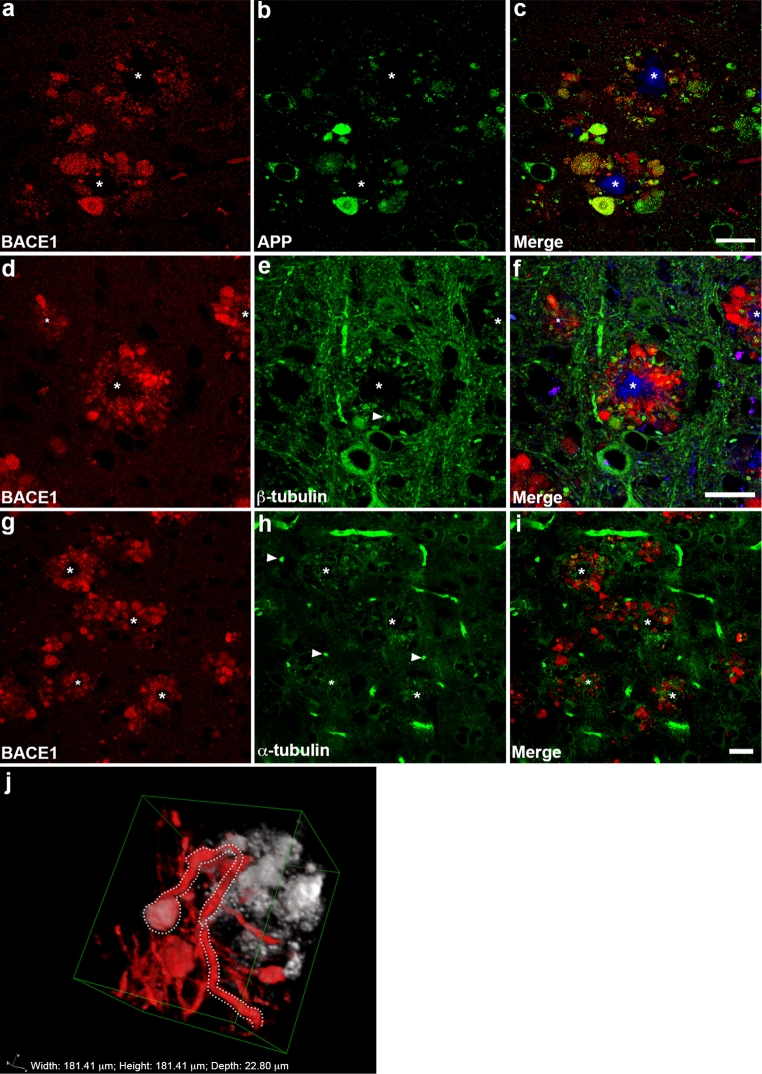
BACE1 co-localizes with APP and neurofilament, but not tubulin, around plaques in the 5XFAD brain. Coronal brain sections from ~6-month-old 5XFAD mice were co-stained with antibodies against BACE1 and either APP, β-tubulin, α-tubulin, or medium neurofilament (NFT160) and imaged by laser scanning confocal microscopy. **a**–**c** BACE1 immunoreactivity (*red*) exhibits variable but significant overlap with that of APP (*green*) within dystrophies around thioflavin S-positive plaques (*blue*), suggesting that APP could be processed by BACE1 in these abnormal structures. In contrast, BACE1 and β-tubulin (**d**–**f**) and α-tubulin (**g**–**i**) immunoreactivities display largely non-overlapping patterns around plaques. **e**, **h** Note that irregular spherical accumulations of tubulin are observed nearby plaques (*arrowheads*). **j** Sequential *z* axis images of BACE1 (*white*) and medium neurofilament (*red*) immunoreactivities were taken and subsequently stacked in Nikon Elements with alpha-blending. BACE1 co-localizes with neurofilament in a dystrophic neurite (*dotted white line*). Importantly, the process of the neurite, immunolabeled for neurofilament, ends in a dystrophy, likely an axon terminal. *Scale bars* **a**–**i**, 20 μm; Dimensions for *box* in **j**: width, 181.41 μm; height, 181.41 μm; depth, 22.80 μm

Previously, we determined that BACE1 levels are elevated in the brains of humans with AD and APP transgenic mice [121]. Moreover, we observed that BACE1 accumulates in synaptophysin-positive structures surrounding the cores of amyloid plaques. However, that study did not determine the precise identity and ultrastructure of the BACE1-containing entities. To accomplish this, we performed immunofluorescence confocal microscopy, conventional EM, and serial section pre-embedding immuno-EM as before, on hippocampal and cortical sections from the brains of the 5XFAD mouse model of AD, which displays memory impairments and development of amyloid plaques [75] that are consistent with human AD pathology. As with wild-type mice, 5XFAD mice exhibited high levels of BACE1 within the hilar region of the dentate gyrus, the infrapyramidal bundle, and stratum lucidum of the hippocampus via immunofluorescence confocal microscopy (Fig. 4a). Moreover, BACE1 immunoreactivity was aberrantly localized to an annulus surrounding each amyloid plaque (Fig. 4d, g), also consistent with our previous findings as well as others [12, 105, 120, 121]. BACE1 immunostaining around plaques generally had a punctate appearance (e.g., Fig. 4g), suggesting vesicular localization, as expected [106]. Importantly, 5XFAD hippocampal sections co-stained with antibodies against APP and BACE1 revealed significant co-localization (Fig. 5a–c). These results demonstrate that the BACE1 enzyme and APP substrate both accumulate in overlapping areas in close proximity to amyloid plaques, precisely where elevated BACE1 cleavage of APP could lead to locally increased Aβ production and exacerbation of amyloid pathology.

Previous studies in both rodents and in humans have shown that many of the structures encircling plaques are dystrophic presynaptic terminals [9, 65, 66, 89, 121], though distended postsynaptic elements may also appear nearby plaques [6]. We have previously shown that many BACE1-positive dystrophies are engorged presynaptic structures in the AD and 5XFAD brain [121]. Using immunofluorescence confocal microscopy, we likewise demonstrated that BACE1 immunoreactivity overlapped that of synaptophysin within many of the neuritic dystrophies adjacent to amyloid plaques (Fig. 4d–f). Conversely, BACE1-positive structures did not co-localize with the somatodendritic marker MAP2 (Fig. 4g–i) [121]. To determine the extent to which this pattern of BACE1 localization in 5XFAD brain recapitulated that in human AD brain, we performed BACE1 and synaptophysin (Fig. 4j–l) or MAP2 (Fig. 4m–o) co-staining of AD hippocampal sections followed by immunofluorescence confocal microscopy. Similar to the 5XFAD brain, BACE1 and synaptophysin displayed significant co-localization surrounding amyloid plaques, while MAP2 did not overlap with BACE1 in the AD brain. These results support our previous work [121] and confirm that 5XFAD mice are faithful models of amyloid-associated BACE1 elevation in AD.

To further characterize the pattern of BACE1 accumulation surrounding amyloid plaques, we co-stained 5XFAD brain sections with anti-BACE1 and antibodies that recognize either neuron-specific class III β-tubulin (Fig. 5d–f) or α-tubulin (Fig. 5g–i), which comprise microtubules. Interestingly, BACE1 showed minimal co-localization with β-tubulin or α-tubulin, suggesting that regions of BACE1 accumulation had reduced levels of microtubules. Moreover, a significant proportion of α- and β-tubulin staining around plaques was concentrated in aberrant spherical, oblate, or ring-like structures (Fig. 5e, h, arrowheads); such structures are unlikely to contain normal bundles of functional microtubules. Given the evidence of abnormal cytoskeletal elements in peri-plaque regions [8], we additionally co-labeled 5XFAD brain sections with antibodies against neurofilament (NFT), a major axonal cytoskeleton component, and BACE1. A 3-dimensional reconstruction of an immunofluorescence confocal microscopy *z*-series showed extensive labeling of axons coursing through brain tissue, with some axons ending as engorged bulbous structures that also contained BACE1 immunoreactivity (Fig. 5j). Thus far, our data indicated that (1) BACE1 localizes to normal mossy fiber terminals of hippocampal region CA3; (2) BACE1 accumulates in dystrophic presynaptic axon terminals surrounding amyloid plaques in the AD and 5XFAD brain; (3) little if any BACE1 localizes to postsynaptic structures, and (4) aberrant microtubule structures are present in regions that lack BACE1.

**Fig. 6:**
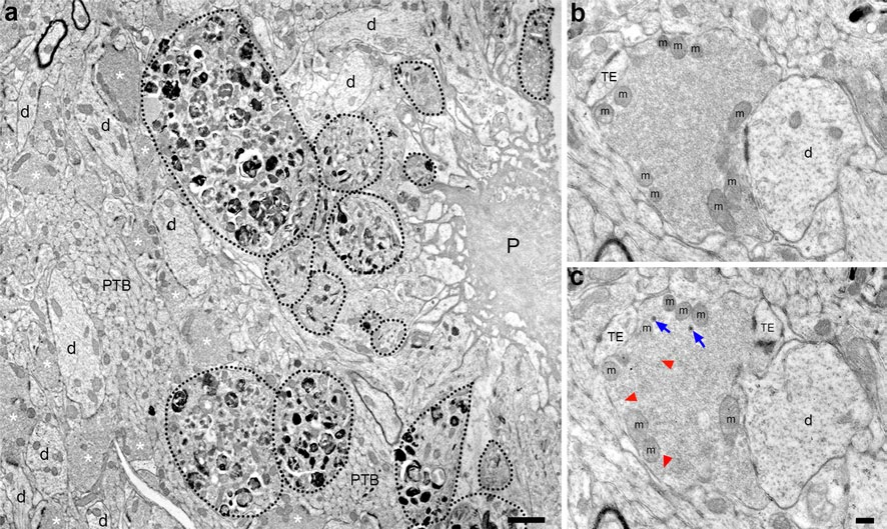
Swollen dystrophic neurites form near amyloid plaques in the 5XFAD brain. **a** Low magnification electron microscope image of a plaque (P) surrounded by dystrophic neurites (*dotted black lines*) in the stratum lucidum of the CA3 hippocampal subregion of a 14-month-old 5XFAD mouse. Note that many of the dystrophic neurites are filled with vesicles that contain electron-dense material. Neighboring dendrites (d), preterminal mossy fiber bundles (PTB), and presynaptic terminals (*white asterisks*) appear normal. **b**, **c** High magnification electron microscope serial images of a single normal appearing mossy fiber terminal from the 5XFAD mouse. The vesicle-rich presynaptic terminal makes synaptic contact with a thorny excrescence (TE) and a dendrite (d) and contains an abundance of small clear vesicles, as well as some large clear vesicles (*red arrowheads*) and dense-core vesicles (*blue arrows*). Mitochondria (m) align near the plasma membrane of the terminal. *Scale bars* **a**, 1 μm; **b**–**c**, 200 nm

To investigate the nature of the BACE1-containing dystrophies at the ultrastructural level, we performed conventional EM and immuno-EM of 5XFAD hippocampal sections, as described above. First, we examined the ultrastructure of normal MFTs in the 5XFAD brain. Despite the abundance of distended neuronal processes surrounding plaques, neighboring MFTs were morphologically comparable to those observed in wild-type and BACE1^−/−^ brains by conventional EM (Fig. 6). These vesicle-rich axon terminals contained numerous small clear vesicles, and to a lesser extent some large clear vesicles and dense-core vesicles (Fig. 6b, c). Dendrites and thorny excrescences protruded into MFTs, making synaptic contacts, and mitochondria aligned along the inside border of the plasma membrane of the axon terminal. To ultrastructurally localize BACE1 in the 5XFAD brain, we turned again to immuno-EM. Light microscopic examination of pre-embedding silver-intensified BACE1 immunogold in the 5XFAD hippocampus revealed BACE1 immunoreactivity in stratum lucidum, the IPB and hilar region of the dentate gyrus (Suppl. Fig. 2a,), which was consistent with fluorescent BACE1 immunolabeling (Fig. 4a). At the EM level, BACE1 immunogold particles were predominantly located within presynaptic compartments in stratum lucidum, with variable signal intensity and distance from active zones within a given axon terminal (Suppl. Fig. 2b–g). These results mirror the presynaptic pattern of BACE1 localization within MFTs of wild-type mice.

**Fig. 7:**
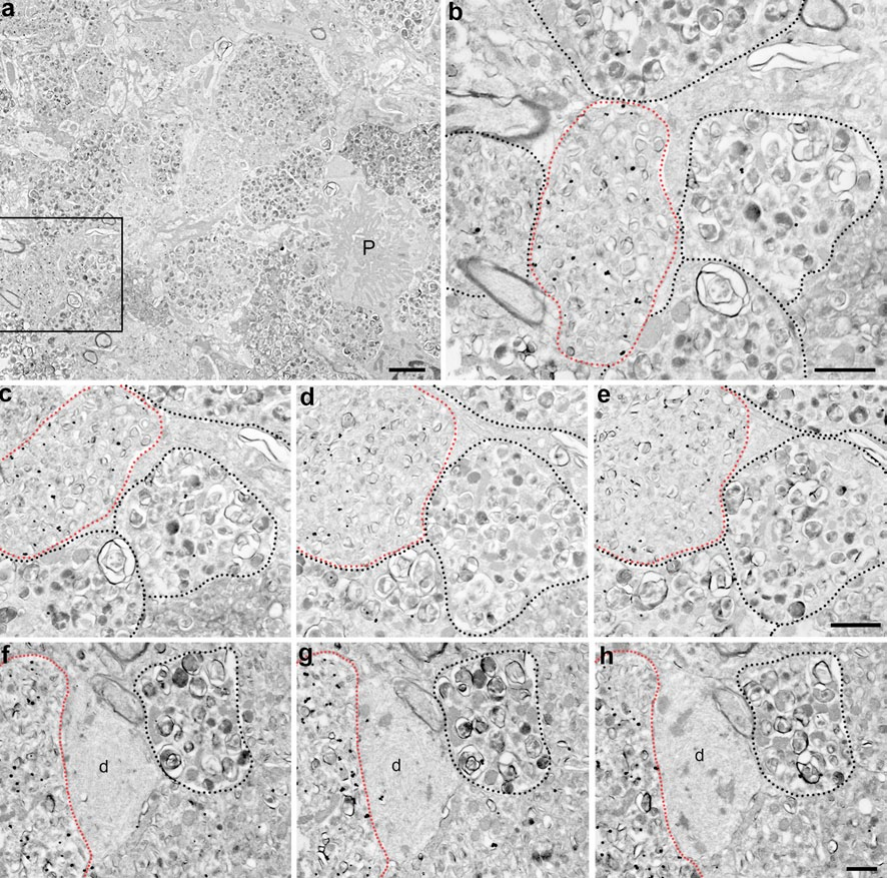
BACE1 is localized within an electron-translucent subtype of plaque-associated dystrophic neurite. Coronal brain sections from 4- to 14-month-old 5XFAD mice were processed for BACE1 pre-embedding silver-intensified ultrasmall immunogold and then serial ultrathin sections of the stratum lucidum and cortex were imaged by electron microscopy. **a** Low magnification image of an amyloid plaque (P) surrounded by dystrophic neurites in the hippocampal CA3 subregion of the 5XFAD brain. **b** Higher magnification of *boxed region* in **a** showing BACE1-positive and BACE1-negative dystrophic neurites. Two types of dystrophic neurites are apparent, which we refer to as Type I (*red dotted line*) and Type II (*black dotted lines*). The different subtypes are distinguishable by the amount of electron density (Type I, less; Type II, more) and the size of vesicular structures (Type I, smaller vesicles; Type II, larger vesicles). Note that BACE1 immunogold labeling is more prominent in Type I dystrophic neurites (*red dotted lines*). **c**–**e** Representative images of serial ultrathin sections of BACE1 immunogold-labeled cortical neuritic dystrophies from the 5XFAD brain, with Type I dystrophic neurites predominantly enriched with BACE1 immunogold particles (*red dotted lines*) adjacent to Type II dystrophic neurites (*black dotted lines*). **f**–**h** Representative serial images of a BACE1-immunopositive Type I dystrophic neurite (*red dotted lines*) adjacent to an aberrant dendrite (d) and Type II dystrophic neurite (*black dotted lines*) within CA3. Note the Type II dystrophic neurite contains very few BACE1 immunogold particles. Close examination of the aberrant dendrite (d) reveals abnormal microtubule organization exhibiting a swirling pattern. *Scale bars* **a**, 2 μm; **b**, 1 μm; **c**–**e**, 200 nm; **f**–**h**, 1 μm

In the electron microscope, distended neuronal processes in the 5XFAD hippocampus were readily apparent at low magnification (Figs. 6a, 7a). These abnormal, swollen neurites were invariably near plaques and appeared analogous to those observed in other AD mouse models [8, 23, 56, 67, 76, 89, 119] as well as human AD brains [45, 66, 72, 92, 100]. Moreover, the 5XFAD neuritic dystrophies were densely packed with lamellar structures of variable morphology and size (Figs. 6a, 7). Dystrophic neurites have been shown to be positive for markers of autophagy [14, 45, 72] and are typically filled with an abundance of autophagic intermediates, as viewed at the EM level [8, 23, 45, 56, 67, 72, 76, 89, 100, 119]. Indeed, many of the lamellar structures in the 5XFAD dystrophies were morphologically similar to autophagosomes observed in human AD and AD mouse models.

Upon further inspection of BACE1 immunogold-labeled 5XFAD sections, ultrastructurally there appeared to be two subtypes of dystrophic neurites that we will refer to as Type I and Type II (Fig. 7). Type I dystrophies contained numerous small round, oval, or irregularly shaped membraneous structures densely packed into an enlarged membrane-bound neurite. Type II dystrophies were distended membrane-bound neurites filled with large vacuolar structures of varying morphologies, many of which resembled autophagosomes with electron-dense centers that were encircled by at least a single or multiple concentric membranes. The amount of electron-dense material varied from one vacuolar structure to another, and in general Type II neurites appeared more electron dense than Type I neurites. The two neuritic subtypes surrounded amyloid plaques in both hippocampus and cortex of the 5XFAD brain. Mitochondria were also present in both subtypes. Although 5XFAD dystrophic neurites generally could be classified as either Type I or Type II, we observed that some dystrophies contained variable mixtures of vesicular structures found in both subtypes, suggesting that a continuum of neuritic morphologies might exist between Type I and Type II neurites. Interestingly, the smaller, less electron-dense vesicles of the Type I neurites were often immunoreactive for BACE1, whereas the larger, more electron-dense vesicles of the Type II neurites exhibited considerably less BACE1 immunogold labeling (Fig. 7). Overall, these findings support our immunofluorescence confocal microscopy results showing variable, non-uniform BACE1 immunosignal intensity in different dystrophic neurites surrounding plaques in the AD and 5XFAD brain (Fig. 4).

**Fig. 8:**
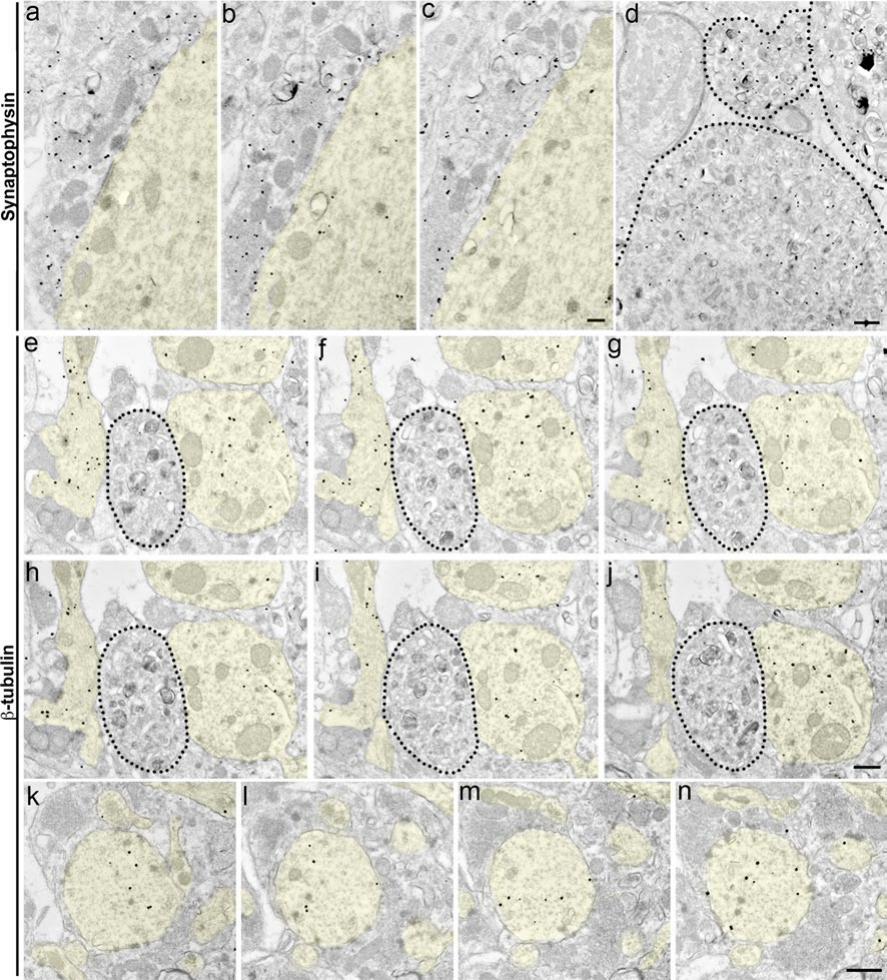
Type I dystrophic neurites are immunoreactive for the presynaptic marker synaptophysin, but not β-tubulin. Coronal brain sections from ~6-month-old 5XFAD mice were processed for pre-embedding silver-intensified ultrasmall immunogold for synaptophysin (**a**–**d**) or β-tubulin (**e**–**n**) and then serial ultrathin sections of the stratum lucidum were imaged by electron microscopy. **a**–**c** Representative serial images of a single normal mossy fiber terminal exhibiting synaptophysin immunogold particle labeling. Note that synaptophysin immunoreactivity is absent in the adjacent postsynaptic dendrite (*yellow shading*). **d** Representative image of Type I dystrophic neurites (*black dotted lines*) that are labeled with synaptophysin immunogold particles, indicating that they are presynaptic structures. **e**–**j** Images of serial ultrathin sections labeled with β-tubulin immunogold particles. Note that dendrites (*yellow shading*) are positive for β-tubulin, while the dystrophic neurite (*black dotted lines*) lacks β-tubulin immunoreactivity. **k**–**n** Other serial images of a representative β-tubulin immunogold labeled dendrite (*yellow shading*). β-Tubulin immunoreactivity is absent from surrounding mossy fiber terminals. *Scale bars* 500 nm

Based on our BACE1-synaptophysin immunofluorescence co-localization results, we hypothesized that the BACE1-positive Type I dystrophic neurites were likely to be presynaptic structures. To explore this possibility, we immunogold-labeled 5XFAD brain sections with an anti-synaptophysin antibody and determined whether morphologically defined Type I dystrophies were synaptophysin-positive. As expected, our electron microscopic analysis revealed enrichment of synaptophysin immunogold particles within normal mossy fiber presynaptic terminals (Fig. 8a–c). Moreover, synaptophysin immunoreactivity was detected within neuritic dystrophies (Fig. 8d), consistent with other published reports [9, 65, 66, 89, 121]. These synaptophysin-positive dystrophies predominantly contained smaller, electron translucent vesicles and therefore resembled BACE1-positive Type-I dystrophic neurites. We additionally labeled 5XFAD sections with a neuron-specific class III β-tubulin antibody. Dystrophic neurites were largely devoid of β-tubulin immunogold particles; however, dendrites were clearly immunopositive for β-tubulin in the 5XFAD brain (Fig. 8e–n). Other reports also have shown that plaque-associated dystrophic neurites lack MAP2 immunoreactivity [8, 89]. Together, these results support the conclusion that the abnormal, swollen structures in the 5XFAD brain were dystrophic presynaptic terminals.

**Fig. 9:**
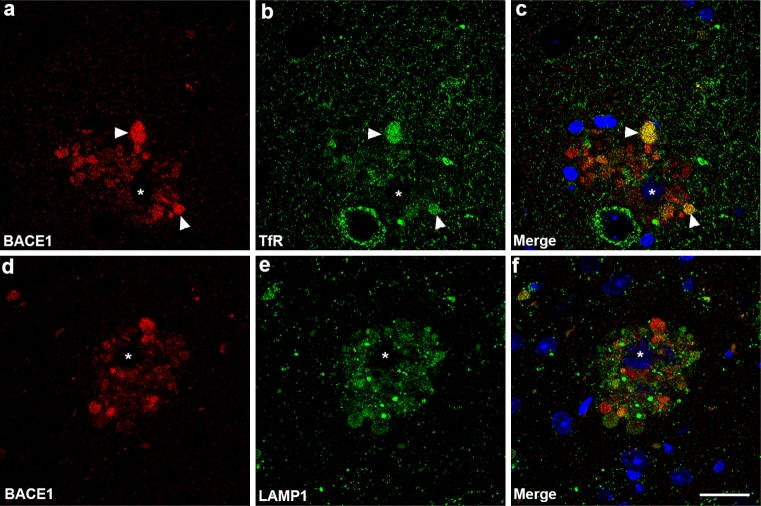
BACE1 co-localizes with the endosomal marker transferrin receptor, but not with the lysosomal marker LAMP1, in dystrophic neurites. Representative images of brain sections from ~6-month-old 5XFAD mice co-stained with BACE1 (*red*) and transferrin receptor (TfR) or LAMP1 (*green*) antibodies and imaged by laser scanning confocal microscopy. **a**–**c** BACE1 exhibits significant co-localization with TfR in endosomes (e.g., *white arrowheads*) within dystrophic neurites surrounding an amyloid plaque (*white asterisk*). **d**–**f** In contrast, BACE1 immunoreactivity occurs in a largely non-overlapping pattern with that of the late endosome/early lysosome marker, LAMP1, in dystrophic neurites that surround an amyloid plaque (*white asterisk*). Note that dystrophic neurites immunopositive for LAMP1 typically display minimal BACE1 immunoreactivity, and vice versa. The reason for this is unknown, but could occur if BACE1 undergoes degradation in lysosomes, thus destroying the BACE1 epitope. *Blue* in **c**, **f** indicates DAPI stain for nuclei and plaque cores. *Scale bar* 20 μm

Next, we wanted to determine the type of vesicles within which BACE1 resides in dystrophic neurites that surround amyloid plaques. Therefore, we performed immunofluorescence confocal microscopy of 5XFAD brain sections to co-localize the late endosome/lysosome marker lysosomal-associated membrane protein 1 (LAMP1), and the early endosome marker transferrin receptor, along with BACE1 (Fig. 9). BACE1 in dystrophic neurites co-localized significantly with transferrin receptor (Fig. 9a–c), suggesting that a large proportion of BACE1 resides within endosomes/endocytic compartments. In contrast, BACE1 exhibited relatively little co-localization with LAMP1 within a given neuritic dystrophy (Fig. 9d–f). Taken together, these data suggest that BACE1 resides primarily within endosomal vesicles, rather than lysosomes, within dystrophic neurites in the 5XFAD brain.

### BACE1 is degraded by lysosomes, not by autophagy

**Fig. 10:**
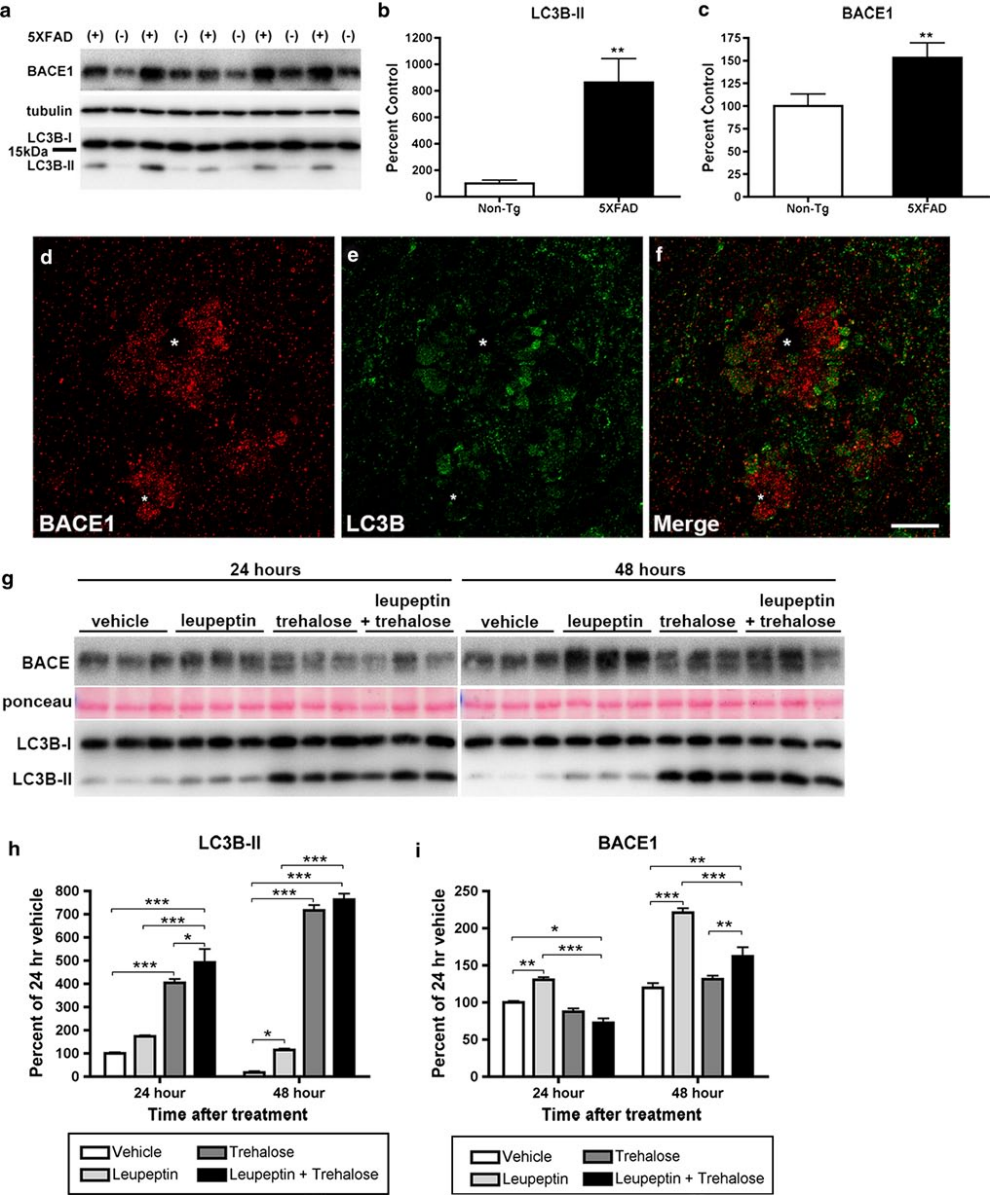
BACE1 is degraded by lysosomes, but not by autophagy. **a**–**f** The autophagy marker LC3B-II is elevated in the 5XFAD brain. **a** Homogenates of individual hippocampi from ~6-month-old 5XFAD (+) and non-transgenic (−) mice were analyzed by immunoblot for BACE1 and LC3B. Note the increased intensity of the LC3B-II band in 5XFAD compared to non-transgenic hippocampi. β-Tubulin was used as a loading control. **b** Densitometric analysis of LC3B-II signal intensity in **a**, normalized to that of LC3B-I, shows ~700 % increase in the LC3B-II:LC3B-I ratio in 5XFAD hippocampi compared to non-transgenic controls. **c** Densitometric analysis of BACE1 signal intensity in **a**, normalized to that of β-tubulin, shows ~50 % increase in BACE1 levels in 5XFAD hippocampi compared to non-transgenic controls (**p < 0.01, *t* test, *n* = 5 for **b**, **c**). **d**–**f** Representative images of brain sections from ~6-month-old 5XFAD mice co-stained with BACE1 (*red*) and LC3B (*green*) antibodies and imaged by laser scanning confocal microscopy. BACE1 and LC3B immunoreactivities largely occur in non-overlapping patterns in dystrophic neurites surrounding amyloid plaques (*white asterisks*). Note that dystrophic neurites immunopositive for LC3B typically display minimal BACE1 immunoreactivity, and vice versa. **g** Primary cortical neuron cultures from wild-type E15.5–16.5 mouse embryos were treated with either vehicle, 20 μM leupeptin (a lysosomal protease inhibitor), 150 mM trehalose (an inducer of autophagy), or leupeptin plus trehalose for 24 or 48 h, and then analyzed for BACE1, LC3B-I and LC3B-II levels by immunoblot. Ponceau staining was used as a loading control. **h** Densitometric analysis of the ratio of LC3B-II to LC3B-I levels in **g** shows a dramatic increase in LC3B-II:LC3B-I with trehalose and leupeptin plus trehalose treatment, demonstrating that trehalose is a strong inducer of autophagy in neurons. In contrast, leupeptin alone causes only a relatively modest elevation of neuronal LC3B-II:LC3B-I ratio. **i** Densitometric analysis of BACE1 levels in **g**, normalized to ponceau, shows a marked increase of BACE1 levels with leupeptin treatment in neurons, while trehalose does not decrease BACE1 levels. These results suggest that BACE1 is not cleared by autophagy but instead is likely degraded in the lysosomal pathway in neurons, at least in culture (*p < 0.05, **p < 0.01, ***p < 0.001 ANOVA, *n* = 3 for **h**, **i**). *Scale bar* **d**–**f**, 20 μm

Thus far, our confocal and electron microscopy results suggested that BACE1 is localized within endocytic vesicles that accumulate in a subtype of dystrophic presynaptic terminal surrounding amyloid plaques. In addition, our EM data, especially in the Type II dystrophies, clearly indicated an accumulation of autophagic intermediates, consistent with EM reports of human AD and other AD mouse models, suggesting elevated autophagy in the 5XFAD brain. To confirm this biochemically, we performed immunoblot analysis for a marker of autophagosomes, LC3B-II [3], in hippocampal homogenates of 6-month-old 5XFAD mice and non-transgenic littermates (Fig. 10). During autophagy, LC3B-I is conjugated with phosphatidylethanolamine to form LC3B-II, which becomes associated with the autophagic membrane and changes from diffuse to punctate intracellular localization [41]. We observed a dramatic increase in the immunoblot signal ratio of LC3B-II to LC3B-I (~9-fold) in 5XFAD hippocampal homogenates (Fig. 10a, b), consistent with the accumulation of autophagic intermediates and a recent report also showing an increased LC3B-II: LC3B-I ratio in an APP transgenic [89]. In addition, confocal microscopy of 5XFAD cortex showed punctate LC3B immunostaining surrounding amyloid plaques in a pattern that exhibited minimal co-localization with BACE1 labeling (Fig. 10d–f), reminiscent of the distribution of BACE1-negative autophagosomes in Type II dystrophic neurites by EM. Immunoblot analysis also revealed that BACE1 levels were significantly increased in the hippocampi of 5XFAD mice compared to non-transgenic littermates (Fig. 10a, c), similar to our previous reports [74, 121]. These elevations of LC3B-II and BACE1 levels by immunoblot and immunofluorescence microscopy corroborate our electron microscopy data demonstrating the accumulation of autophagosomes and BACE1 in dystrophic neurites, and may reflect either impaired clearance of autophagosomes, increased induction of autophagy, or a combination of both in the 5XFAD brain.

A major goal of our study was to gain insight into the mechanism of BACE1 elevation in AD. Given our results that both autophagy and BACE1 were increased in the 5XFAD brain, we tested the hypothesis that BACE1 is a substrate of autophagy; if so, BACE1 elevation could arise from failure of autophagy to clear BACE1. To determine whether BACE1 is degraded by autophagy, we treated primary murine cortical neurons with two inducers of autophagy, rapamycin [73] and trehalose [90], and the lysosomal protease inhibitor leupeptin, followed by immunoblot analysis for BACE1 and LC3B. Leupeptin was used to test the alternative hypothesis that BACE1 is degraded by lysosomes. While rapamycin failed to induce autophagy in primary neurons (data not shown), as reported previously [54], trehalose treatment for either 24 or 48 h dramatically increased the LC3B-II:LC3B-I ratio (~7-fold by 48 h) compared to vehicle (Fig. 10g–h), an increase similar to that observed in the 5XFAD brain (Fig. 10a, b). Although leupeptin treatment also increased LC3B-II:LC3B-I ratio, supporting previous work that inhibition of lysosomal function results in decreased clearance of autophagosomes [48, 53, 101], it did so to a much lesser extent than trehalose (Fig. 10g–h). Combined leupeptin plus trehalose treatment did not markedly increase LC3B-II:LC3B-I ratio beyond that of trehalose alone. Importantly, immunoblot analysis revealed that, compared to vehicle, trehalose treatment did not reduce BACE1 levels in primary neurons (Fig. 10g, i), suggesting that BACE1 is not degraded in the autophagic pathway. Interestingly, BACE1 levels in primary neurons treated with leupeptin were increased to over 200 % of vehicle by 48 h (Fig. 10g, i), a BACE1 elevation similar in magnitude to that observed in the 5XFAD (Fig. 10a, c) and the AD brain. A similar increase in BACE1 level was obtained by treating neurons with bafilomycin, an inhibitor of endosome/lysosome acidification (data not shown). These results are consistent with previous reports showing that BACE1 is trafficked to the lysosome for degradation [51, 103]. Combined leupeptin plus trehalose treatment produced a small but significant increase of BACE1 level by 48 h compared to trehalose alone (Fig. 10g, i). Taken together, our findings support the conclusion that BACE1 is not cleared by autophagy, but instead is degraded in lysosomes, at least in primary cortical neurons in culture. Further, these cell culture data suggest that decreased lysosomal function could lead to both elevations of BACE1 and autophagy in AD.

## Discussion

### BACE1 in the normal presynaptic terminal

BACE1 is required for Aβ generation. Thus, it is a promising AD therapeutic target (reviewed in [44, 107]). However, the numerous BACE1 substrates [30, 55, 123] and complex phenotypes of BACE1 null mice suggest that the inhibition of BACE1 for AD may not be free of mechanism-based toxicity. Thus, knowledge of BACE1 physiological functions is necessary to predict and potentially avert side effects of BACE1 inhibitor drugs.

One poorly understood question is the role of BACE1 in the brain, the target organ of BACE1 inhibitors. Although BACE1^−/−^ mice indicate that BACE1 is involved in memory [57, 77, 78], myelination [37, 113], seizure [33, 39, 50], axon guidance [13, 32, 85], emotions [57], schizophrenia [91] and vision [11], the mechanisms of these BACE1 null neurological phenotypes are not fully understood. The subcellular localization of BACE1 in the brain may provide important clues as to the roles of BACE1 in the CNS and the molecular and cellular bases of BACE1 functions.

Thus, we have investigated BACE1 cerebral localization at both light and electron microscopic levels. To our knowledge, this is the first study to determine the subcellular localization of endogenous BACE1 in neurons of the brain. Our previous work suggested that BACE1 is concentrated in presynaptic terminals, especially in mossy fibers of the stratum lucidum in hippocampal CA3 [121]. Using mono-specific anti-BACE1 antibodies, we performed immunofluorescence confocal microscopy and determined that BACE1 is highly localized within synaptophysin-positive puncta in large mossy fiber terminals (giant boutons) of CA3. Little if any BACE1 is localized to MAP2-positive somatodendritic postsynaptic sites in the hippocampus, although BACE1-positive puncta are found in neuronal soma (TGN, endosomes).

Immuno-EM revealed that BACE1 is localized to vesicles within mossy fiber terminals. BACE1-positive vesicles were located near synaptic active zones, although this was not always the case. The specific localization of BACE1 to membranous vesicular structures within presynaptic terminals suggests an important but as yet undetermined function of BACE1 substrate processing at the synapse. As with our immunofluorescence confocal microscopy, only background BACE1 immunogold was observed postsynaptically. Taken as a whole, our results demonstrate that the presynaptic terminal is the principal site of BACE1 localization in the brain.

The function of BACE1 in the presynaptic terminal is currently unknown. In tissue culture cells, overexpressed BACE1 mainly resides in acidic compartments (TGN, endosomes) where BACE1 substrate cleavage occurs. Endosomes within presynaptic terminals have been reported [42, 87, 110] and although they have not been extensively studied, evidence suggests that presynaptic endosomes are involved in neurotransmitter vesicle recycling [10, 28, 35, 82, 86]. Further investigation of presynaptic endosomes may reveal the role of BACE1 within this compartment, which may reflect the need for proteolytic processing of one or more BACE1 substrates at or near the synapse. Three BACE1 substrates that may be cleaved in the presynaptic terminal are Na_v_β_2_, CHL1, and neuregulin, which play a role in axon depolarization [46], neurite outgrowth/axon guidance [32] and myelination [37, 113], respectively. Presumably, BACE1-cleaved Na_v_β_2_ and neuregulin fragments would be trafficked from the presynaptic terminal to the axon via retrograde transport to exert their effects. However, BACE1 presynaptic localization argues that a different substrate(s) is processed by BACE1 in the terminal where it may perform a function required specifically at that location.

One such BACE1 substrate is APP, which has been hypothesized to play a role in neuroprotection [29], cell adhesion [99, 117], neurite outgrowth (reviewed in [104]), synapse formation or maintenance [80], as well as regulating synaptic transmission [43]. APP is transported to the neuronal terminal [52, 97] where it is likely processed by BACE1 and γ-secretase to generate secreted APP ectodomain (APPsβ), APP C-terminal fragment (β-CTF), and Aβ [40, 64, 96, 106, 118]. It is possible that APPsβ released at the terminal may function to protect or maintain synaptic contacts. An intriguing alternative hypothesis is that Aβ itself may be involved in synaptic function or neurotransmission. Neuronal stimulation causes secretion of Aβ at the terminal [18], a process that requires endocytosis [17]. Other studies suggest that Aβ may regulate glutamatergic synaptic transmission [16] and facilitate LTP [83, 84, 115, 116] at low endogenous Aβ concentrations normally released at the terminal. Thus, BACE1 in the presynaptic terminal may serve to process APP into Aβ for the latter’s presumptive role in synaptic function.

Another interesting BACE1 substrate that may function at the presynaptic terminal is CHL1 [32, 55, 123]. Importantly, BACE1^−/−^ axon guidance defects in the hippocampus and olfactory bulb phenocopy axon targeting errors observed in CHL1^−/−^ mice [31, 32, 68]. Moreover, CHL1 is processed by BACE1, and CHL1 and BACE1 co-localize in primary neuron growth cones and in presynaptic terminals in hippocampus and olfactory bulb [32], suggesting that BACE1 cleavage of CHL1 is necessary for proper axon guidance. The action of other BACE1 substrates at the terminal could also explain BACE1 presynaptic localization. Numerous BACE1 substrates have been identified [30, 55, 123] and others will likely be discovered in the future. Additional studies will be necessary to validate putative BACE1 substrates in vivo and thereby clarify the role of BACE1 in the presynaptic terminal.

### BACE1 in the dystrophic presynaptic terminal surrounding the amyloid plaque

Several studies report that BACE1 levels are elevated in AD brains [27, 34, 62, 121]. These results raise two intriguing questions: (1) Does BACE1 elevation exacerbate AD pathogenesis? (2) What mechanism is responsible for BACE1 elevation? These questions have potential implications for AD mechanisms and novel therapeutics. To gain insight into the second question, we performed immunofluorescence confocal and immunogold electron microscopy to determine where cerebral BACE1 accumulates in the 5XFAD transgenic mouse model of amyloid pathology. BACE1 immunofluorescence confocal microscopy and immuno-EM of 5XFAD brain sections showed endogenous BACE1 localization within normal CA3 mossy fiber presynaptic terminals with occasional BACE1 labeling near active zones. In addition, BACE1 accumulates within presynaptic dystrophies that surround amyloid plaques within the hippocampus and cortex. Importantly, APP exhibited a high degree of co-localization with BACE1 in 5XFAD presynaptic dystrophic neurites, suggesting the intriguing possibility that BACE1 processing of APP might occur in these dystrophies to exacerbate Aβ generation and plaque formation. APP has been reported to localize within dystrophic neurites of AD brain [7, 8, 19, 22, 89]. In addition, we have shown here and in a previous study [121] that BACE1 co-localizes with synaptophysin, but not MAP2, in dystrophic neurites surrounding plaques in human AD brain in a pattern that is similar to that seen in 5XFAD brain. We conclude that 5XFAD mice recapitulate the pattern of BACE1 accumulation in plaque-associated dystrophies observed in human AD. Thus, investigation of BACE1 elevation in 5XFAD mice should provide valuable insight into the formation and progression of amyloid plaques in AD.

We observed different subtypes of dystrophic neurites in the 5XFAD brain, which we termed Type I and Type II, that were distinguishable based on the size, degree of electron density, and multi-lamellar nature of membrane-bound (autophagic intermediate-like) structures within a given neurite. Generally, Type I dystrophic neurites were less electron dense and contained smaller membraneous structures, while Type II dystrophies were more electron dense and contained larger, multi-lamellar structures. BACE1 immuno-EM signal was highest in Type I dystrophies that contain smaller, less electron-dense vesicles. Type II dystrophic neurites, which exhibit large multi-lamellar autophagosomes, had much lower BACE1 immunogold labeling. Although the identity of BACE1-positive vesicles in Type I dystrophies is unknown, we suspect they are endosomes based on the high degree of co-localization of BACE1 and transferrin receptor by immunofluorescence confocal microscopy. Poor co-localization of BACE1 with LAMP1 and LC3B allows us to exclude lysosomes and autophagosomes as primary organelles of BACE1 accumulation, respectively. These data concur with our immuno-EM results showing lack of BACE1 localization to multi-lamellar autophagosomes. We speculate that Type I and Type II dystrophies represent a continuum of the disease process, whereby Type I represents an earlier stage of disease. This notion is supported by EM studies that characterize dystrophic neurites at different stages of degeneration in AD mouse models [1, 23] and human AD [109], the latter in which two types of dystrophies were described having either large globoid APP and chromogranin-immunopositive or tau-immunopositive morphologies. The former may represent an early stage of dystrophy, while the latter are neurofibrillary tangle (NFT)-containing structures at a late stage of degeneration. 5XFAD mice do not have NFTs and therefore lack this late-stage dystrophic neurite. However, 5XFAD dystrophies may be related to early-stage globoid APP and chromogranin-positive AD dystrophic neurites, thus fitting with the general consensus that APP transgenic mice model an earlier phase of AD.

### Roles of autophagy, lysosomes, and microtubule transport in BACE1 accumulation in AD

Dystrophic neurites are positive for markers of autophagy [14, 72] and at the ultrastructural level contain multi-lamellar autophagosomes [8, 23, 45, 71, 72, 89, 100, 119] observed in 5XFAD Type II dystrophies. Additionally, 5XFAD hippocampi had an elevated LC3B-II:LC3B-I ratio, indicating increased autophagy consistent with accumulation of autophagosomes by EM and concurring with elevated LC3B-II:LC3B-I ratio in another APP transgenic [89]. The increased markers of autophagy correlate with elevated BACE1 levels in 5XFAD hippocampus, although both BACE1 immuno-EM and BACE1-LC3B co-localization by confocal microscopy suggest that BACE1 does not accumulate in autophagosomes.

BACE1 accumulation in endosomes of 5XFAD presynaptic dystrophic neurites suggests three potential mechanisms: (1) BACE1 is degraded by autophagy, but an earlier step of the autophagic pathway is impaired (e.g., fusion of endosomes with autophagosomes); (2) BACE1 is degraded by lysosomes, but an earlier step of the lysosomal pathway is impaired (e.g., lysosomal acidification or maturation); (3) BACE1 clearance requires microtubule transport, but microtubules are dysfunctional (e.g., failure of BACE1 transport back to the soma). To gain initial insights into these mechanisms, we investigated the roles of autophagy and the lysosomal pathway in BACE1 clearance in cultured primary neurons. Previous studies reported that axonal dystrophy could result from inhibition of lysosomal proteolysis [4, 5, 58, 59]. Treatment of primary neurons with the lysosomal protease inhibitor leupeptin resulted in elevated BACE1 levels and a small increase in the LC3B-II:LC3B-I ratio, the latter of which indicates reduced autophagosome clearance. These results support earlier work suggesting that BACE1 is degraded in lysosomes [51, 103]. In contrast, inducing autophagy in primary neurons with trehalose resulted in a large increase in LC3B-II:LC3B-I ratio, but had no effect on BACE1 level. If autophagy cleared BACE1 in neurons, then BACE1 should significantly decrease following trehalose treatment. Hypothetically, BACE1 could be an autophagy substrate under other conditions of autophagy induction; however, we find this possibility unlikely. Hence, BACE1 does not appear to be a target of autophagy, at least in primary neurons. Rather, our data indicate that BACE1 accumulation may result from impaired lysosomal degradation of BACE1.

Taken together, our in vitro and in vivo results indicate that BACE1 accumulates in endosomes within presynaptic dystrophic neurites surrounding plaques, suggesting that BACE1 degradation could be reduced due to decreased BACE1 flux through the endosomal–lysosomal pathway. Although the 5XFAD brain exhibits robust accumulation of autophagosomes and increased LC3B-II, autophagy does not appear to be directly involved in BACE1 clearance. The evidence for this is the following: (1) BACE1 co-localizes with synaptophysin and transferrin receptor but not MAP2, LAMP1 or LC3B, indicating that BACE1 accumulates in presynaptic endosomes but not lysosomes or autophagosomes; (2) BACE1 immunogold labels small electron translucent vesicles, not large electron-dense multi-lamellar autophagosomes; and (3) lysosomal protease inhibition elevates neuronal BACE1, but autophagy induction does not reduce BACE1.

The reason for reduced endosomal–lysosomal BACE1 flux is unclear, although aberrant accumulation of α- and β-tubulin in dystrophic neurites implies that dysfunctional microtubules could play a role. Lysosome maturation requires vesicles laden with lysosomal proteases that are derived from the Golgi apparatus in the soma. If microtubule-based axon transport is impaired by amyloid, deficient protease levels in presynaptic lysosomes may ensue resulting in reduced protein degradation. Although speculative, this model would provide a plausible mechanism for increased BACE1 levels in dystrophies near plaques. Similarly, organelle accumulation in dystrophic neurites has been postulated to arise from impaired axon transport [70]. In addition, AD brains show reduced levels of dynein and kinesin, molecular motors critical for axoplasmic transport [69]. Loss of retrograde transport in dystrophies could also contribute to accumulation of autophagosomes and BACE1-positive vesicles in axon terminals, causing neurites to swell as vesicles and mitochondria amass within. The molecular nature of the plaque-related toxic agent and mechanism responsible for neuritic dystrophy and BACE1 accumulation are unclear, but likely involve Aβ, given the intimate association of dystrophies with plaques. Alternatively, dystrophic neurite formation may involve reticulon/Nogo proteins (reviewed in [81]), since transgenic mice that overexpress reticulon-3 form plaque-independent neuritic dystrophies [38, 94, 95]. Future work should elucidate mechanisms of dystrophic neurite pathogenesis.

In summary, our study provides the first conclusive evidence, as shown by electron microscopy, that BACE1 resides predominantly within normal and dystrophic presynaptic terminals in wild-type and AD mouse model brain, consistent with our immunofluorescence microscopy of human AD. Presynaptic BACE1 localization suggests that BACE1 substrate processing has important consequences for axon terminal function. BACE1 and APP accumulate in presynaptic dystrophies surrounding amyloid plaques, implying a feed-forward mechanism of Aβ generation that may exacerbate AD pathogenesis. Deficient lysosomal degradation, but not impaired autophagy, of BACE1 could cause BACE1 accumulation in presynaptic endosomes. Although the molecular mechanism of BACE1 elevation around plaques is enigmatic, evidence of abnormal tubulin accumulation implies dysfunctional microtubule-based transport. Other mechanisms are possible, such as impaired lysosomal acidification or enzymatic activity [58]. Future studies to elucidate mechanisms of BACE1 elevation in dystrophies may provide insights into potential therapeutic avenues to reduce BACE1 levels and ameliorate AD.

## Electronic supplementary material

Below is the link to the electronic supplementary material.

Supplementary material 1 (TIFF 5009 kb)

Supplementary material 2 (TIFF 12973 kb)

Supplementary Fig 1 BACE1 immunogold particles label mossy fibers in the hippocampus. Coronal brain sections from 4 to 12 month old BACE1^+/+^ **a**, **c** and BACE1^−/−^ **b**, **d** mice were incubated with a rabbit monoclonal anti-BACE1 antibody and processed for immunofluorescence **a**, **b** or pre-embedding silver-intensified ultrasmall immunogold **c**, **d** and then imaged by light microscopy. **a**, **b** Laser scanning confocal microscope images of BACE1 immunofluorescence signal (*red*) in BACE1^+/+^ and BACE1^−/−^ hippocampus. Note the absence of BACE1 signal in the BACE1^−/−^ hippocampus, demonstrating that the anti-BACE1 antibody is mono-specific. **c** Light microscope image of a resin-embedded BACE1^+/+^ mouse hippocampal section that underwent pre-embedding silver-enhanced immunogold labeling for BACE1, showing the same pattern of immunoreactivity as in **a**. **d** Silver-intensified BACE1 immunogold signal is lacking in BACE1^−/−^ hippocampus. Hilar region of the dentate gyrus (H), infrapyramidal bundle (IPB), stratum lucidum (SL). *Scale bar* **a**–**d**, 200 μm.

Supplementary Fig 2 Mossy fiber terminals that are not associated with plaques have normal BACE1 localization and ultrastructure. **a** Resin-embedded silver-enhanced BACE1 immunogold-labeled hippocampal section from a 14 month old 5XFAD mouse imaged by light microscopy reveals BACE1 immunoreactivity in the dentate gyrus hilus (H), infrapyramidal bundle (IPB), and stratum lucidum (SL) of the CA3 subregion. BACE1 immunostaining surrounding plaques within the hippocampus and cortex is also evident (*black arrows*). **b**, **c**–**e**, **f**–**g** High magnification single **b** or serial (**c**–**e**, **f**–**g**) electron microscope images through individual normal mossy fiber terminals from the 5XFAD brain. BACE1 immunogold particles are enriched within presynaptic terminals. In some cases, gold particles localize adjacent to synaptic active zones (*red arrowheads*). Postsynaptic regions (*yellow shading*) including thorny excrescences (TE) and dendritic shafts (d), lack BACE1 immunoreactivity. In many cases, mitochondria (m) cluster near the plasma membrane of the terminal. *Scale bars* in **e**: **a**, 200 μm; **b**–**g**, 200 nm.
