# Supplementary figures and images for: The Alzheimer’s β-secretase BACE1 localizes to normal presynaptic terminals and to dystrophic presynaptic terminals surrounding amyloid plaques
Source: Acta Neuropathol. 2013 Jul 3;126(3):329–52. doi: 10.1007/s00401-013-1152-3 (PMC3753469; doi:10.1007/s00401-013-1152-3)

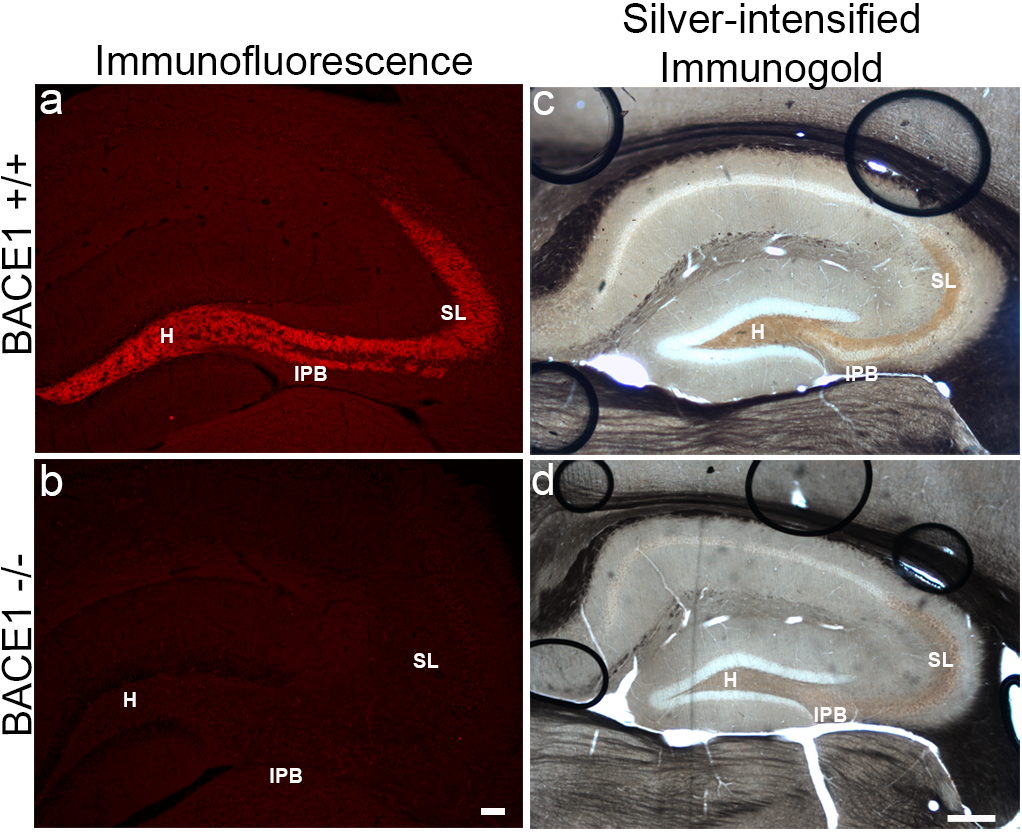

Supplement: Supplementary file 1 — Supplementary material 1 (TIFF 5009 kb) [file 401_2013_1152_MOESM1_ESM.tif]

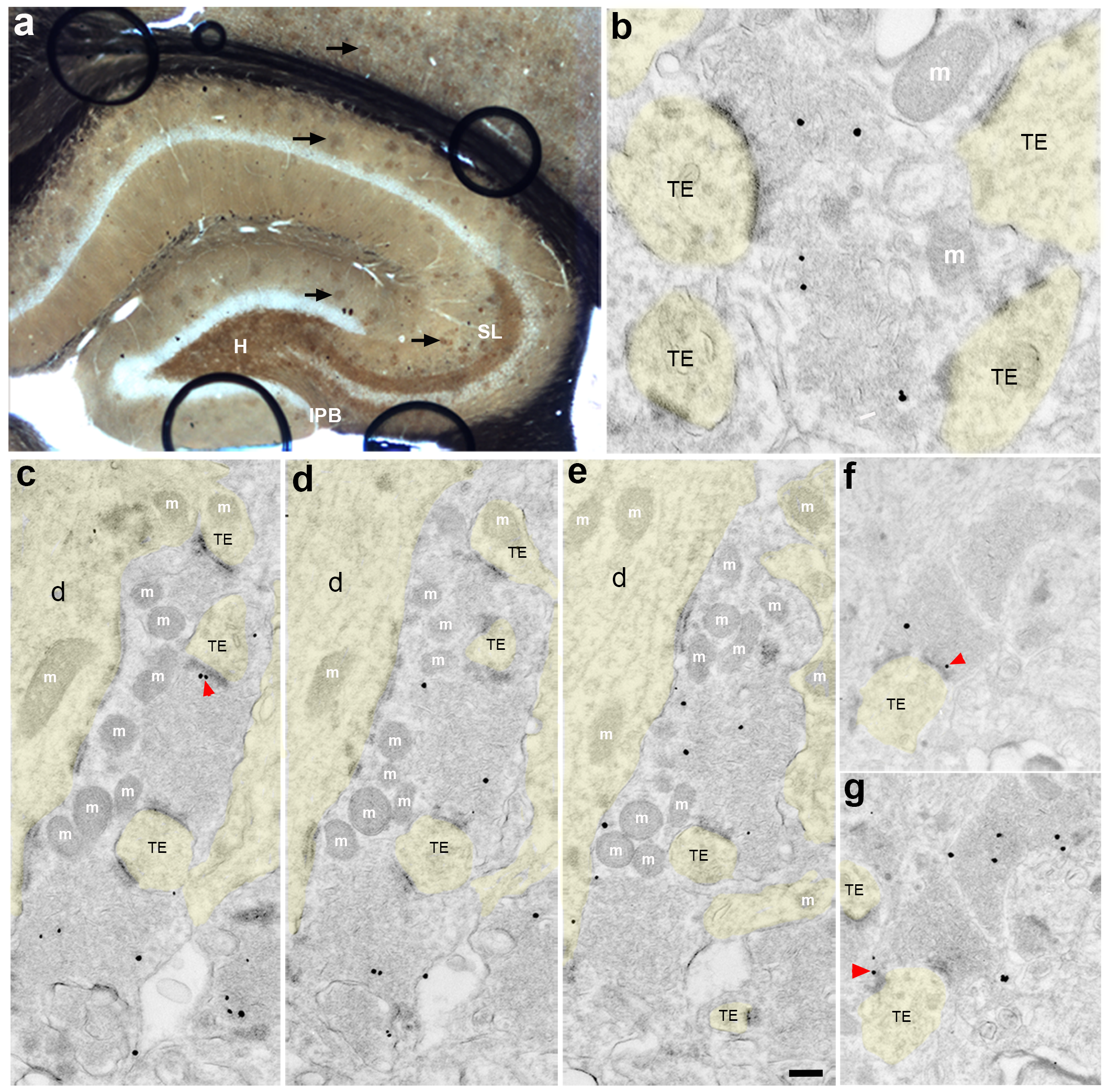

Supplement: Supplementary file 2 — Supplementary material 2 (TIFF 12973 kb) [file 401_2013_1152_MOESM2_ESM.tif]
